# Changes in enterovirus serotype constituent ratios altered the clinical features of infected children in Guangdong Province, China, from 2010 to 2013

**DOI:** 10.1186/s12879-016-1690-0

**Published:** 2016-08-09

**Authors:** Hong-Tao Zhou, Yong-Hui Guo, Man-Jun Chen, Yu-Xian Pan, Lin Xue, Bin Wang, Shao-Hua Tao, Nan Yu

**Affiliations:** 1 Laboratory of Emerging Infectious Diseases and Division of Laboratory Medicine, Zhujiang Hospital, Southern Medical University, No. 253 Gong ye da dao zhong, Guangzhou, 510282 People’s Republic of China; 2 Department of Paediatrics, Zhujiang Hospital, Southern Medical University, Guangdong, 510282 People’s Republic of China; 3 Department of Clinical Laboratory, Hainan Provincial People’s Hospital, No. 19 Xiuhua road, Haikou, 570311 People’s Republic of China

**Keywords:** Enterovirus, Hand, Foot and mouth disease, Clinical features, Serotype

## Abstract

**Background:**

Enterovirus (EV)-related hand, foot, and mouth disease/herpangina (HFMD/HA) has been prevalent in Guangdong Province, China, since 2010.

**Methods:**

Clinical data for EV-related HFMD/HA inpatients admitted to the Department of Paediatrics of Zhujiang Hospital from 2010 to 2013 were retrospectively reviewed. The corresponding EV serotypes were also determined by reverse transcription-polymerase chain reaction or BLAST analysis of the sequenced partial lengths of the viral protein1/5′-untranslated region.

**Results:**

A total of 867 eligible inpatients admitted during 2010–2013 were included in the study. Of these, the serotype of the responsible EV was successfully identified in 824 cases. The incidence of enterovirus 71 (EV71) infection amongst pediatric HFMD/HA inpatients decreased dramatically from 55.5 % in 2010 to 8.1 % in 2013, with a similar decrease recorded for coxsackievirus A16 (CVA16). However, the incidence of non-EV71/CVA16 infection increased from 30.0 % in 2010 to 83.8 % in 2013. We noted that the types of infection caused by different EV serotypes varied: EV71 was responsible for 100 % of the paralysis cases (26/26), 84.6 % of the deaths (11/13), and 84.1 % of cases with severe central nervous system involvement (SCNSI) (74/88); echovirus contributed to 16.4 % of the deaths (2/13) and 4.4 % of the SCNSI cases; and coxsackievirus accounted for only 2.2 % of the SCNSI cases (2/90). The clinical features of HFMD/HA cases varied greatly during the time period examined, with drastic changes in the hospitalization rates (45.1, 63.7, 36.4, and 19.1 % for 2010, 2011, 2012, and 21013, respectively), mortality rates (2.3, 0.9, 2.5, and 0.0 %, respectively), paralysis (5.1, 1.2, 5.4, and 0.0 %, respectively), SCNSI (16.8, 7.1, 12.7, and 2.2 %, respectively), and acute respiratory infection (21.1, 22.0, 45.9, and 59.0 %, respectively).

**Conclusions:**

The incidences of infection caused by different EV serotypes, along with the clinical features of HFMD/HA cases, changed drastically in Guangdong Province, China, from 2010 to 2013, with the biggest changes observed in 2013. The changed constituent ratios of the different EV serotypes might therefore be responsible for the differences in the observed clinical features of HFMD/HA during this period.

**Electronic supplementary material:**

The online version of this article (doi:10.1186/s12879-016-1690-0) contains supplementary material, which is available to authorized users.

## Background

Many enterovirus (EV) serotypes, including EV71, coxsackievirus A (CVA2, 4, 5, 7, 9, 10, 16), and coxsackievirus B (1, 2, 3, 4, 5), have been reported to cause hand, foot and mouth disease/herpangina (HFMD/HA) in humans. Of these, EV71 and CVA16 have traditionally been the two most common serotypes in mainland China [1–6]. In addition to fever and a rash, respiratory infection, hepatic injury, myocardial injury, meningitis, encephalitis, and acute flaccid paralysis are observed in EV-related HFMD/HA [7–9]. Different serotypes of EV mutually overlap in disease causation, and no serotype is assigned to a particular disease. However, some serotypes are more associated with particular diseases, such as EV71, which is associated with neurogenic pulmonary edema, brainstem encephalitis, and myelitis in young children [10–13]. The constituent ratios of EV serotypes change without cessation on a global basis, and monitoring their associated changes in the constituent ratios of circulating EV serotypes and their associated diseases is essential for public health and efficient allocation of medical resources.

Mainland China is heavily affected by EV-related HFMD/HA, which has caused millions of infections and hundreds of deaths annually since 2009 [14]. Guangdong, a southern province of China, is one of the most severely affected regions [15–17]. Zhujiang Hospital is an officially assigned tertiary hospital for treating HFMD patients in Guangzhou (the provincial capital of Guangdong Province), as well as referral patients from other regions of Guangdong Province. Using a retrospective analysis of clinical information regarding HFMD/HA patients admitted to Zhujiang Hospital with an ascertained EV serotype, the current study aimed to elucidate the serotype constituent evolution of EV, and to explore its effect on HFMD/HA in Guangdong Province, China, between 2010 and 2013.

## Methods

### Patients and clinical data

A retrospective study was performed using pediatric patients with HFMD/HA admitted to Zhujiang Hospital between January 2010 and December 2013. All cases were from Guangzhou, or were referrals from other regions of Guangdong Province. Pediatric patients fitting the following criteria were included in the study: real-time fluorescence reverse transcription (RT)-polymerase chain reaction (PCR) evidence of positive EV; signs of eruptions or fever; and younger than 16 years of age. To reduce interference from other diseases, inpatients with a medical history of on-going neurological symptoms, congenital heart disease, or obvious bacterial infection were excluded. The clinical records of each inpatient were retrospectively itemized by two researchers, and then confirmed by pediatric clinicians. Patient privacy was maintained as the cases remained anonymous. The study was approved by the Ethics Committee of Zhujiang Hospital (ratification no.: ZJYY-2013-YXJYZX-001).

### Stool sample collection and molecular analysis

A stool specimen was collected from each patient as part of standard care, and RNA was extracted from a 140-μL aliquot using a QIAamp Viral RNA Mini Kit (Qiagen, Hilden, Germany) within 1 day of collection. Some of the extracted RNA was used for immediate PCR analysis, while the remaining stool specimen and RNA were stored at −80 °C. Extracted RNA samples were used for real-time fluorescence RT-PCR and/or amplification of partial lengths of the viral protein 1/5′-untranslated region (VP1/5′-UTR). EV71 and CVA16 were jointly identified using EV71 and CVA16 EV kits (Shanghai ZJ Bio-Tech Co., Ltd, Shanghai, China). The serotypes of non-EV71 and non-CVA16 strains were initially determined by amplification of a partial length of VP1, with further amplification of a partial length of the 5′-UTR if necessary for clarification. The VP1/5′-UTR fragments were amplified as described previously [18, 19]. The products were sequenced by the Huada Gene Company (Shenzhen, China), and then analyzed using the BLAST program against sequences available in the GenBank database to identify the serotype.

### Clinical definitions

Central nervous system involvement (CNSI) cases were defined as having two or more of the following: vomiting, irritability, startle reflex, convulsion, changed muscular tension, decreased tendon reflex, headache, neck stiffness, or any sign of altered consciousness. Paralysis and central nervous system lesions (on neuroimaging) were categorized as severe central nervous system involvement (SCNSI). Acute respiratory infection (ARI) was diagnosed by the presence of rhinorrhea and/or cough, and patients with pneumonia or bronchopneumonia were categorized as having a severe ARI. Acute myocardial injury was diagnosed based on laboratory evidence of elevated cardiac troponin I (cTnI), elevated creatine kinase-MB (CK-MB), and anelevated CK-MB/CK ratio. The following values were considered the threshold over which the readings were considered elevated: cTnI >0.04 μg/L, CK-MB/CK >0.25, CK-MB >44 U/L (2–36 month), CK-MB >37 U/L (37–72 month), and CK-MB >31 U/L (>73 month). Patient with an alanine aminotransferase level of >40 U/L was deemed as having acute hepatic injury. Diarrhoea was defined as presenting changed consistency of stool or frequency of defecation ≥3 times/day.

### Statistical analysis

Statistical analysis was performed using SPSS version 13.0 software (SPSS Inc., Chicago, IL, USA). The differences in the categorical variables were analyzed by chi-square test. All tests were two-sided, and *p* < 0.05 was considered statistically significant.

## Results

**Fig. 1:**
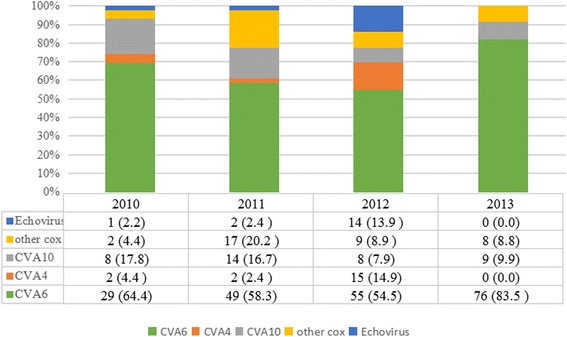
Constituent ratios infections with CVA4, CVA6, CVA10, other coxsackieviruses or Echovirus in 320 genotying cases between 2010 and 2013. Cox: coxsackieviruses

A total of 2394 EV-related HFMD/HA patients were admitted to the Department of Paediatrics of Zhujiang Hospital during the study period, consisting of 926 inpatients and 1468 outpatients, and 824 inpatients were ascertained infected EV serotype by PCR and/or sequencing. Numbers of inpatients infected with EV71, CVA16 or non-EV71/CVA16 were 390, 127 and 409, respectively; while those of outpatients were 308, 259 and 901, correspondingly. Of the 926 inpatients, 867 were deemed eligible and were included in the current study (2010 (212), 2011 (314), 2012 (201), 2013 (140)), and the numbers of eligible inpatients infected with EV71, CVA16 or non-EV71/CVA16 included to illustrate the clinical features of HFMD/HA were 384, 120 and 363, respectively. The EV serotypes were successfully ascertained from 320/363 inpatients with non-EV71/CVA16 infection, of which the numbers of cases identified by partial VP1 and 5′-UTR sequencing were 295 and 25, respectively. Amongst the 320 identified EV serotypes, CVA6, CVA4, CVA10, echoviruses, and other coxsackieviruses accounted for 209 (65.3 %), 19 (5.9 %), 39 (12.2 %), 17 (5.3 %), and 36 (11.3 %) of the cases, respectively. CVA6 was responsible for more than half of the non-EV71/CVA16 cases each year during the study period as follows: 64.4 % (2010), 58.3 % (2011), 54.5 % (2012), and 83.5 % (2013) (Fig. 1). Of the 17 sporadic cases of echovirus, 14 were admitted in 2012, with the remaining three patients admitted in 2010 and 2011.

### Significant changes occurred in the constituent ratios of EV serotypes in Guangdong Province between 2010 and 2013

**Fig. 2:**
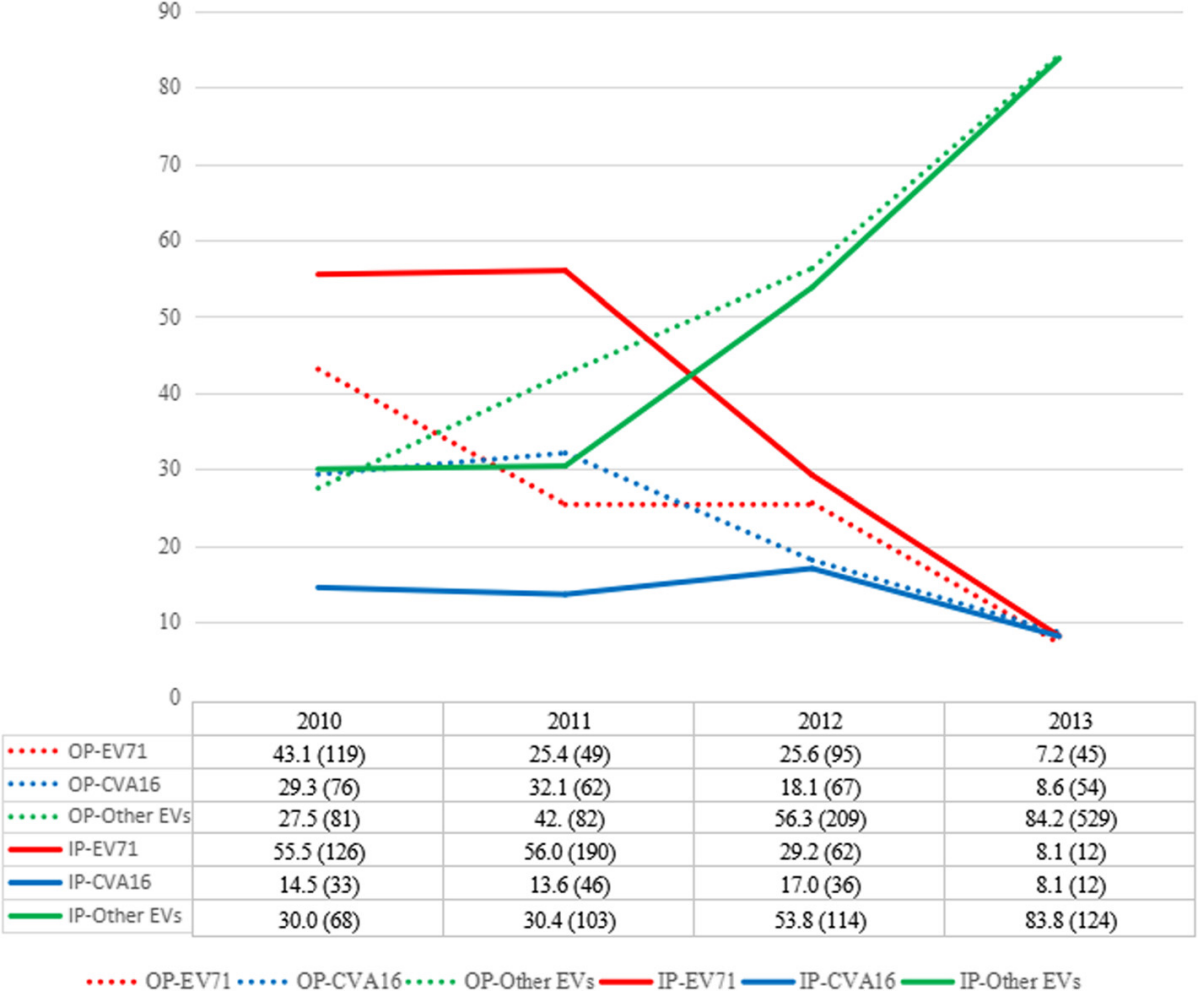
Constituent ratios of children infected with EV71, CVA16, or non-EV71/CVA16 admitted to Zhujiang Hospital between 2010 and 2013. Non-EV71/CVA16: non-enterovirus 71 and non-coxsackievirus A16; IP: inpatient; OP: outpatient; EV71: enterovirus 71; CVA: coxsackievirus A

The constituent ratios of inpatients infected with EV71, CVA16, and non-EV71/CVA16 changed greatly between 2010 and 2013 (Fig. 2). The incidence of EV71-infected inpatients decreased from 55.5 % in 2010 to 8.1 % in 2013, with the incidence of CVA16 infection dropping from 14.5 % in 2010 to 8.1 % in 2013. In contrast, the incidence of non-EV71/CVA16 infection increased from 30.0 % in 2010 to 83.8 % in 2013. Figure 2 shows similar changes in the constituent ratios of outpatients infected with EV71, CVA16, and non-EV71/CVA16. Statistially significant differences were observed in the constituent ratios of EV serotypes in outpatients amongst these years (2011 vs 2012 (*p* = 0.029), others (*p* < 0.01)), and statistially significant differences also observed in inpatients amongst these years (*p* < 0.05), except for 2010 vs 2011 (*p* =0.966).

### Clinical features of HFMD/HA infection changed during the study period

**Fig. 3:**
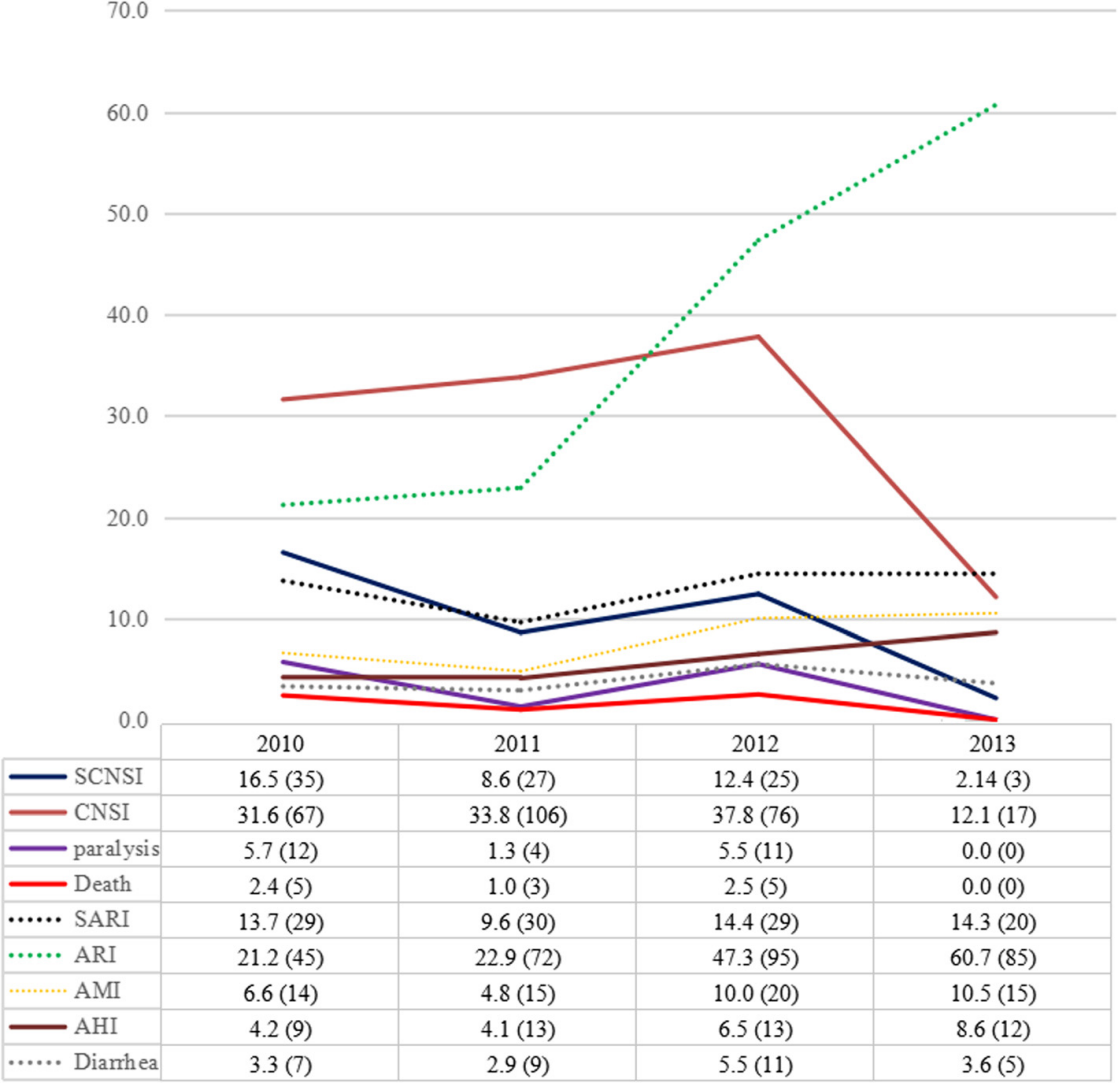
Evolution of the clinical features of HFMD/HA inpatients admitted to Zhujiang Hospital between 2010 and 2013. SCNSI: severe central nervous system involvement; HFMD/HA: hand, foot, and mouth disease/herpangina; CNSI: central nervous system involvement; ARI: acute respiratory infection; SARI: severe acute respiratory infection; AMI: acute myocardial injury; AHI: acute hepatic injury. Title: the relationship of enterovirus serotype constituent ratios and the clinical features of infected children. We observed the phenomneon of obvious change of clinical features of children infected with enterovirus and a notable change of enterovirus serotype constituent ratios in Guangdong province, China during 2010–2013, which suggested that change of enterovirus serotype constituent ratios might be responsible for the phenomenon of obvious change of clincial features of children infected with enterovirus

The clinical features of children with HFMD/HA varied greatly over the study period. The hospitalization rate for HFMD/HA cases decreased from 63.7 % in 2010 to 19.1 % in 2013, while the rates of mortality, paralysis, SCNSI, and CNSI fluctuated drastically between 2010 and 2013 (Fig. 3). Additionally, ARI gradually replaced CNSI as the leading complication of HFMD/HA (Fig. 3). The risk of HFMD/HA sharply declined in 2013, with decreasing incidences of CNSI and SCNSI and no occurrences of paralysis or death.

### The risks of severe complications of HFMD/HA varied depending on EV serotype

Of the 824 cases with an ascertained EV serotype (EV71 (*n* = 384, 46.6 %), coxsackievirus (*n* = 423, 51.3 %), and echovirus (*n* = 17, 2.1 %)), the mortality rates and neurological complications were disproportionately high in infections caused by EV71 and echoviruses, while severe complications were relatively low in cases caused by coxsackievirus. EV71 was responsible for 100 % of the paralysis cases (26/26), 84.6 % of the deaths (11/13), and 82.2 % of the cases of SCNSI (74/90), echovirus was responsible for 16.4 % of the deaths (2/13) and 4.4 % of the cases of SCNSI, while coxsackievirus was responsible for only 2.2 % of the cases of SCNSI (2/90), without causing death or paralysis.

## Discussion

This retrospective study demonstrated that the constituent ratios of EV serotypes in Guangdong Province, China, changed significantly between 2010 and 2013. EV71 was the predominant serotype at the beginning of the study period, but was less prevalent by 2013, with the reverse trend observed for CVA6. By 2013, EV71 infections accounted for less than 10 % of cases, whereas non-EV71/CVA16 serotypes caused more than 80 % of infections (Fig. 2). The overwhelming majority of non-EV71/CVA16 infections were caused by CVA6, indicating that CVA6 was the predominant serotype in Guangdong Province in 2013. Two previous studies also confirmed that CVA6 had become the dominant serotype in Guangdong during late 2012 and 2013 [17, 20], with similar reports from other regions of China in 2013 [21, 22]. CVA6 also replaced EV71 as the dominant serotype in other areas of the Asia-Pacific region, such as Taiwan, Thailand and Janpan in recent years [23–25]. The underlying mechanisms driving the shift of EV serotype constituent in an area maybe caused by the declining number of susceptible population caused by previous dominated serotype circulation and genetic mutation of certain EV serotype gaining edges on circulation.

Our data also showed that the drastically changed constituent ratios of the EV serotypes fundamentally altered the clinical features associated with HFMD/HA in Guangdong Province during the same time period. Overall, the hospitalization rate declined substantially, from 45.1 % in 2010 to 19.1 % in 2013, with the rates of SCNSI, paralysis, and death also declining, apart from a rebound in 2012. The incidence of ARI increased over the 4-year period, and became the most common complication of HFMD/HA in 2012 and 2013 (Fig. 3). The risk of severe complications of HFMD/HA significantly deceased in 2013, with only three inpatients developing SCNSI, and no cases of paralysis or death. This decline was predominantly caused by the decreased constituent ratio of EV71 infections and the increased prevalence of coxsackievirus infection, which causes less severe clinical features in HFMD/HA patients. In agreement with our results, EV71 was repeatedly reported to be responsible for most of the cases resulting in death, encephalitis, or paralysis in HFMD epidemics in the Asia-Pacific region [10–13], whereas the two most common coxsackievirus serotypes, i.e., CVA6 and CVA16, were generally mild with low incidences of SCNSI and death in children [1, 25–27]. Low incidence of diarrhea in HFMD/HA inpatients was observed in each year, which is somehow akin to previous studies [28, 29].

Echovirus was shown to match the capacity of EV71 for causing death and SCNSI (the 17 echovirus infections contributed to 16.4 % of deaths and 4.4 % of the cases of SCNSI), although its relatively low constituent ratio (2.1 %) reduced its influence on the clinical features of HFMD/HA in Guangdong Province over the study period. The two deaths were infected with Echo 2 and Echo 3, respectively; while the four SCNSI cases infected by Echo 2 (2 cases), Echo 3 (1 case) and Echo 1 (1 case) (data not shown). However, a cluster of echovirus infections contributed to the increase in mortality rates and SCNSI in 2012. Therefore, we concluded that the reduced prevalence of EV71 and the increase in coxsackievirus infections were responsible for the changes in clinical features associated with HFMD/HA between 2010 and 2013. In accordance with our results, other countries or regions that experienced a declined threat of HFMD/HA also documented a decrease in EV71 infections and an increase in coxsackievirus infections.

Because this study constitutes a retrospective analysis of patients admitted to an officially designated HFMD hospital that treats patients from the provincial capital and referrals from other regions of Guangdong Province, there is an inherent bias in the study results. Additionally, bias might be generated by uneven patient distribution from the different districts, which might affect the degree to which the clinical features and serotype constituent ratios for EV-related HFMD/HA in Guangdong Province between 2010 and 2013 are represented.

## Conclusions

Our study demonstrated that EV71 was the predominant serotype in Guangdong Province at the beginning of the study period, but had declined in prevalence by 2013. In comparison, the incidence of CVA6 infections significantly increased over the same period, with CVA6 becoming the dominant serotype in 2013. Additionally, our data suggested that the changed constituent ratios of the EV serotypes might have influenced the clinical features of HFMD/HA infection in Guangdong Province, China, from 2010 to 2013.

## Abbreviations

ALT, alanine amino transferase; ARI, acute respiratory infection; CA, coxsackievirus A; CK, creatine kinase; CK-MB, creatine kinase-MB; CNSI, central nervous system involvement; cTnI, cardiac troponin I; EV, enterovirus; HFMD/HA, hand-foot-mouth disease/herpangina; non-EV71/CVA16, non-enterovirus 71 and non-coxsackievirus A16; RT-PCR, reverse transcription-polymerase chain reaction; SCNSI, severe central nervous system involvement; UTR, untranslated region; VP1, viral protein 1

## Additional files

Additional file 1: Sequence data of non-EV71/CVA16 enterovirus isolated from HFMD/HA inpatients admitted to Zhujiang hospital between 2010 to 2013. (XLSX 40 kb) Additional file 2: Demographical and clinical information of HFMD/HA patients admitted to Zhujiang hospital between 2010-2013. (XLS 4462 kb)
